# Bismuth Selenide Nanostructured Clusters as Optical Coherence Tomography Contrast Agents: Beyond Gold-Based Particles

**DOI:** 10.1021/acsphotonics.1c01504

**Published:** 2022-02-07

**Authors:** Jingke Yao, Tamara Muñoz-Ortiz, Francisco Sanz-Rodríguez, Emma Martín Rodríguez, Dirk H. Ortgies, José García Solé, Daniel Jaque, Riccardo Marin

**Affiliations:** † Nanomaterials for Bioimaging Group (nanoBIG), Departamento de Física de Materiales, Facultad de Ciencias, Universidad Autónoma de Madrid, C/ Francisco Tomás y Valiente 7, Madrid 28049, Spain; ‡ Nanomaterials for Bioimaging Group (nanoBIG), Instituto Ramón y Cajal de Investigación Sanitaria, Hospital Ramón y Cajal, Ctra. De Colmenar Viejo, Km. 9,100, Madrid 28034, Spain; § Nanomaterials for Bioimaging Group (nanoBIG), Departamento de Biología, Facultad de Biología, Universidad Autónoma de Madrid, C/ Darwin 2, Madrid 28049, Spain; ∥ Nanomaterials for Bioimaging Group (nanoBIG), Departamento de Física Aplicada, Facultad de Ciencias, Universidad Autónoma de Madrid, C/ Francisco Tomás y Valiente 7, Madrid 28049, Spain

**Keywords:** bismuth selenide, photon scattering, optical coherence tomography, microwave synthesis, topological insulator

## Abstract

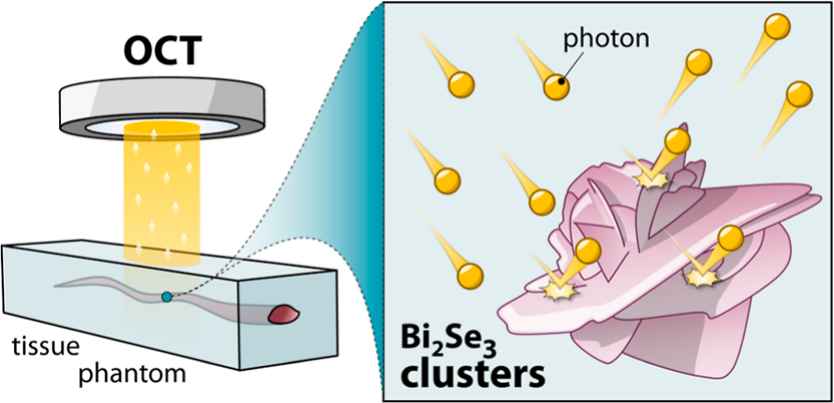

Optical coherence tomography (OCT) is an imaging technique currently used in clinical practice to obtain optical biopsies of different biological tissues in a minimally invasive way. Among the contrast agents proposed to increase the efficacy of this imaging method, gold nanoshells (GNSs) are the best performing ones. However, their preparation is generally time-consuming, and they are intrinsically costly to produce. Herein, we propose a more affordable alternative to these contrast agents: Bi_2_Se_3_ nanostructured clusters with a desert rose-like morphology prepared via a microwave-assisted method. The structures are prepared in a matter of minutes, feature strong near-infrared extinction properties, and are biocompatible. They also boast a photon-to-heat conversion efficiency of close to 50%, making them good candidates as photothermal therapy agents. In vitro studies evidence the prowess of Bi_2_Se_3_ clusters as OCT contrast agents and prove that their performance is comparable to that of GNSs.

## Introduction

Optical coherence tomography (OCT) is emerging as a powerful, minimally invasive diagnostic tool used in clinical practice to obtain anatomical, molecular, and functional images at the ex vivo and in vivo levels. Dermatology,^1−4^ ophthalmology,^5−8^ dentistry,^9−12^ and cardiology^13−16^ are some of the fields that can benefit the most from the use of this imaging technique, which, in its simplest formulation, relies on photon scattering by different tissue components.

To increase the imaging potential of OCT, several contrast agents have been proposed.^17−20^ Among them, plasmonic nanoparticles made of gold are the staple, owing to the strong photon scattering featured by these contrast agents at the probing wavelength used in commercially available OCT instruments—mainly falling in the near-infrared (NIR) range. As a result, gold-based plasmonic nanoparticles such as gold nanoshells (GNSs)^21−23^ and gold nanorods^24−26^ are generally employed as positive contrast agents in OCT studies, with GNSs providing the highest contrast in OCT scans.^27,28^ However, their synthesis is labor- and time-intensive, entailing successive steps of SiO_2_ core growth and coating with gold layers. The expensiveness of noble metals is another shortcoming of these contrast agents, making other nanosystems that could be produced in a shorter time and with reduced costs highly desirable.

In this context, bismuth selenide (Bi_2_Se_3_) nanomaterials are a notable alternative to gold-based contrast agents. Bi_2_Se_3_ is a topological insulator: a family of materials that feature conductive states at the surface while behaving as insulators at their core.^29^ This configuration closely resembles that of GNSs, where the SiO_2_ core is the insulator, and the gold layer is the conductive part. Indeed, similarly to GNSs, Bi_2_Se_3_ nanomaterials exhibit extinction spectra with broad, extended features deep in the NIR, making them well suited to acting as OCT contrast agents. Bismuth also has a large X-ray absorption cross section; hence, nanomaterials based on this metal are suitable contrast agents for other imaging techniques such as computed tomography and angiography. In addition, Bi_2_Se_3_ has been proven biocompatible in several in vitro and in vivo studies.^30−32^

**Scheme 1:**
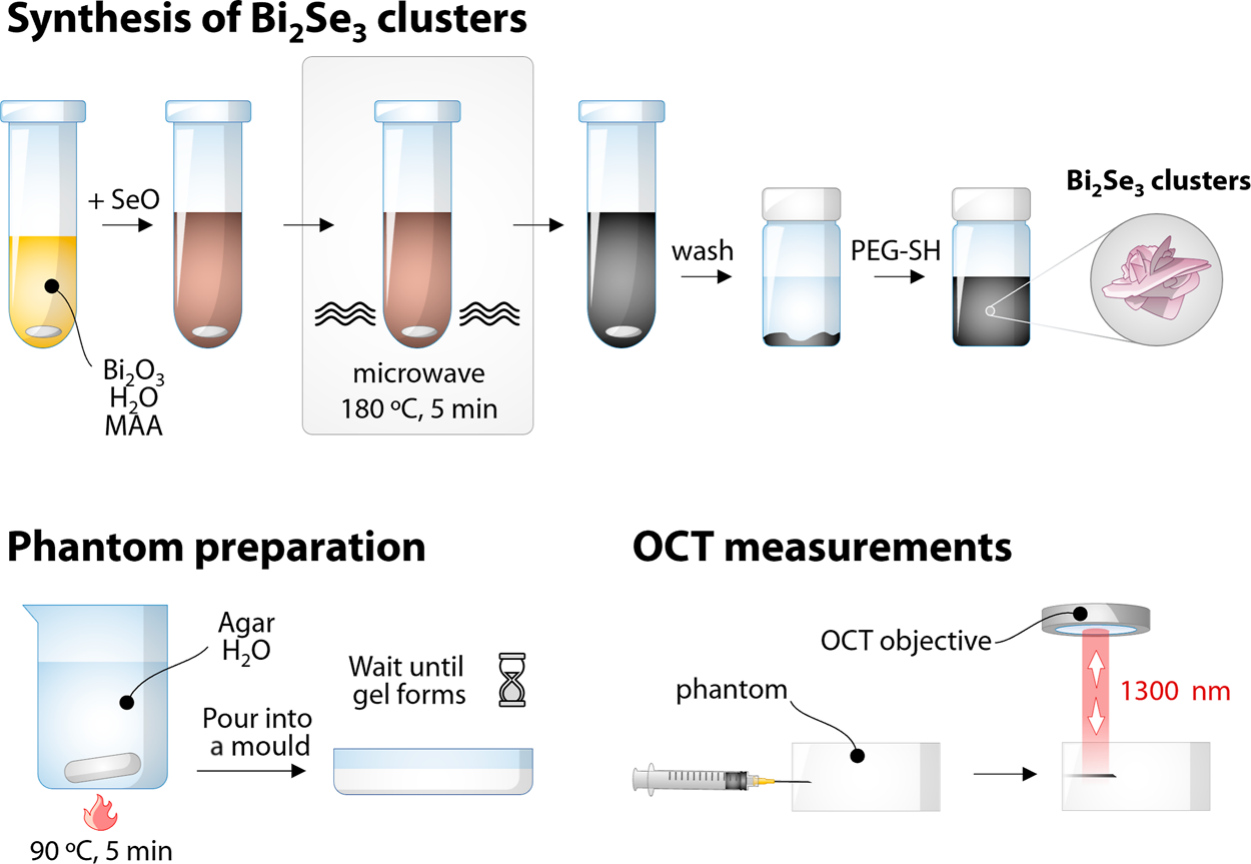
Workflow of the Proposed Study: Microwave-Assisted Synthesis of Bi_2_Se_3_ Nanostructured Clusters and Their Workup, Tissue Phantom Preparation, and OCT Measurements on Phantoms

Moving from these considerations, in this study, we propose an alternative to GNSs as contrast agents for OCT in the form of inexpensive and easy-to-prepare Bi_2_Se_3_ nanostructured clusters. These structures have a desert rose-like morphology and are prepared directly in water via rapid microwave-assisted synthesis (Scheme 1). In vitro tests show no appreciable cytotoxicity of the clusters, supporting their use in the biological context. The topological insulator nature of Bi_2_Se_3_ clusters endows the system with strong extinction capabilities in the NIR range, with roughly half of the impinging photons being scattered. These optical properties featured by the developed clusters ensure strong contrast in OCT images, with performance rivalling that featured by commercially available GNSs.

## Experimental Section

### Chemicals

Bismuth oxide (Bi_2_O_3_, Aldrich, 99.999%), selenium oxide (SeO_2_, Aldrich, 99.8%), mercaptoacetic acid (MAA, Aldrich, 98+%), thiol polyethylene glycol (PEG-SH, MW = 2000, Aldrich), agar powder (Fisher Chemical), Intralipids (Sigma, 20% in water), isopropyl alcohol, and deionized water. All chemicals were used as received.

### Synthesis of Bi_2_Se_3_ Nanostructured Clusters

The reaction was conducted using a CEM Discover 2.0 microwave (MW) reactor. In a 10 mL glass reaction vessel, 46.6 mg (0.1 mmol, for a total of 0.2 mmol Bi^3+^) of Bi_2_O_3_ was added along with 2 mL of MAA and 3.4 mL of deionized water. The formation of metal thiolates (Bi-MAA) was promoted by sonicating the mixture for 1 min at room temperature, until an optically clear, bright yellow solution was obtained. Subsequently, 0.6 mL of a 0.5 M aqueous solution of SeO_2_ (0.3 mmol Se^2−^) was swiftly injected into the Bi-MAA solution under stirring at 1200 rpm. The mixture gradually turned dark brown, and it was stirred at room temperature for 5 min. The vessel was then placed in the MW reactor and the mixture was subjected to heating at 180 °C (heating rate of 20 °C/min) for 5 min. The crude product was precipitated by means of centrifugation (15 min at 3820 rcf) followed by redispersion in 4 mL of deionized water. The particles were precipitated by the addition of 10 mL of ethanol and centrifuged again. The process was repeated three times, and the final product was redispersed in 5 mL of distilled water and stored at 9 °C.

### Surface Modification with Polyethylene Glycol

One milliliter of the Bi_2_Se_3_ cluster dispersion was transferred to a 1.5 mL centrifuge tube along with 12 mg of PEG-SH. The dispersion was sonicated for 5 min and then vortexed for another 2 min. The PEGylated Bi_2_Se_3_ clusters were centrifuged at 30,000 rcf for 10 min at 5 °C. The pellet was redispersed in 1 mL of water. This procedure was repeated two more times, and the particles were finally dispersed in the aqueous medium of choice [deionized water or phosphate-buffered saline (PBS 1×)] and stored at 9 °C.

### Tissue Phantom Preparation

Fifty milliliters of deionized water was introduced into a 100 mL Erlenmeyer flask, which was then introduced into an oil bath preheated at 90 °C. After 5 min, 625 mg of agar powder (1.25% in weight) was introduced, along with 2.5 mL of Intralipids. The mixture was kept under stirring (700 rpm) for approximately 1 h and then poured into a crystallizing dish, covered with paper, and allowed to cool to room temperature until the gel set. The gel was unmolded, sliced, and stored in the fridge for further use.

### OCT Imaging

OCT measurements were conducted using a spectral-domain (SD) instrument (Thorlabs Telesto OG-1300) with a maximum working wavelength of 1300 nm (range 1250–1380 nm), mounting a LSM03 scan lens with a working distance of 25.1 mm, having an axial scan rate of up to 92 kHz, and an axial resolution in water of 4.9 μm, with a maximum imaging depth of 2.5 mm.

### Characterization

The Bi_2_Se_3_ clusters were imaged on carbon-coated copper grids using a JEM1400 Flash (JEOL) transmission electron microscope operating at 80 kV acceleration voltage and a Hitachi S-3000N scanning electron microscope working at 20 kV after drop casting the sample onto a carbon tape-coated support. The hydrodynamic size and ζ-potential were obtained at 25 °C, using a Malvern Zetasizer Nano ZS90 (Malvern) with a detection angle of 173° and an equilibration time of 120 s. The optical extinction spectra were recorded at room temperature using an UV–vis–NIR spectrophotometer (PerkinElmer Lambda 1050) using a 3 nm step. Infrared spectra were obtained in the transmission mode using a IRSpirit Fourier transform IR (FTIR) spectrometer (Shimadzu) in the 450–4000 cm^–1^ range with 4 cm^–1^ resolution by preparing KBr tablets containing 1% wt of the analyzed material. Powder X-ray diffraction (PXRD) measurements were performed using a Rigaku-D/max-γB diffractometer working in the Bragg–Brentano geometry (θ–2θ) with a step of 0.03° in the 20–60° range. X-ray photoelectron spectroscopy (XPS) was performed using a VG Escalab 220i-XL spectrometer equipped with a hemispherical analyzer, applying a twin anode X-ray source. The binding energy was calibrated by reference to the C 1s peak.

### Heat Conversion Efficiency Determination

The photon-to-heat transduction capability of the Bi_2_Se_3_ clusters was evaluated according to the method introduced by Roper et al.^33^ A 1 cm optical path cuvette was filled with 0.8 mL of either water or a Bi_2_Se_3_ cluster dispersion and irradiated using two different lasers (790 and 980 nm). The temperature during irradiation was recorded using a thermocouple inserted into the cuvette. The acquisition of the heating–cooling curves was performed three times for each set of measurements.

### Cytotoxicity Tests

The HeLa human cervical epithelial cell line was grown in Dulbecco’s modified Eagle’s medium (Gibco, Paisley, Scotland, UK) supplemented with fetal calf serum (10%, Gibco) and 0.5% of antibiotics [penicillin G (10,000 U/mL) and streptomycin sulfate (10,000 mg/mL) (Gibco)]. Cells were grown in a Thermo Scientific Midi 40 CO_2_ incubator (Thermo Fisher Scientific Inc.) with a 5% CO_2_ atmosphere, a 95% relative humidity, and a constant temperature of 37 °C.

The viability of HeLa cells exposed to Bi_2_Se_3_ clusters was analyzed using the 3-(4,5-dimethylthiazol-2-yl)-2,5-diphenyltetrazolium bromide (MTT) assay.^34^ Twenty-four hours after appropriate treatments with Bi_2_Se_3_ clusters, an MTT solution was added to each well at a concentration of 0.5 ng/mL, and the plates were incubated at 37 °C for 2 h. The resulting formazan crystals were dissolved by the addition of dimethyl sulfoxide and absorbance was measured at 540 nm. The cell viability was estimated as a percentage relative to the mean absorption obtained from control cells (not incubated with the Bi_2_Se_3_ clusters; 100% viability).

### Statistical Analysis

The quantitative data and the sample size of the cell viability results were expressed as mean ± standard deviation and numbers, respectively. Excel from Microsoft Office suite was the software used for statistical analysis.

## Results and Discussion

**Figure 1:**
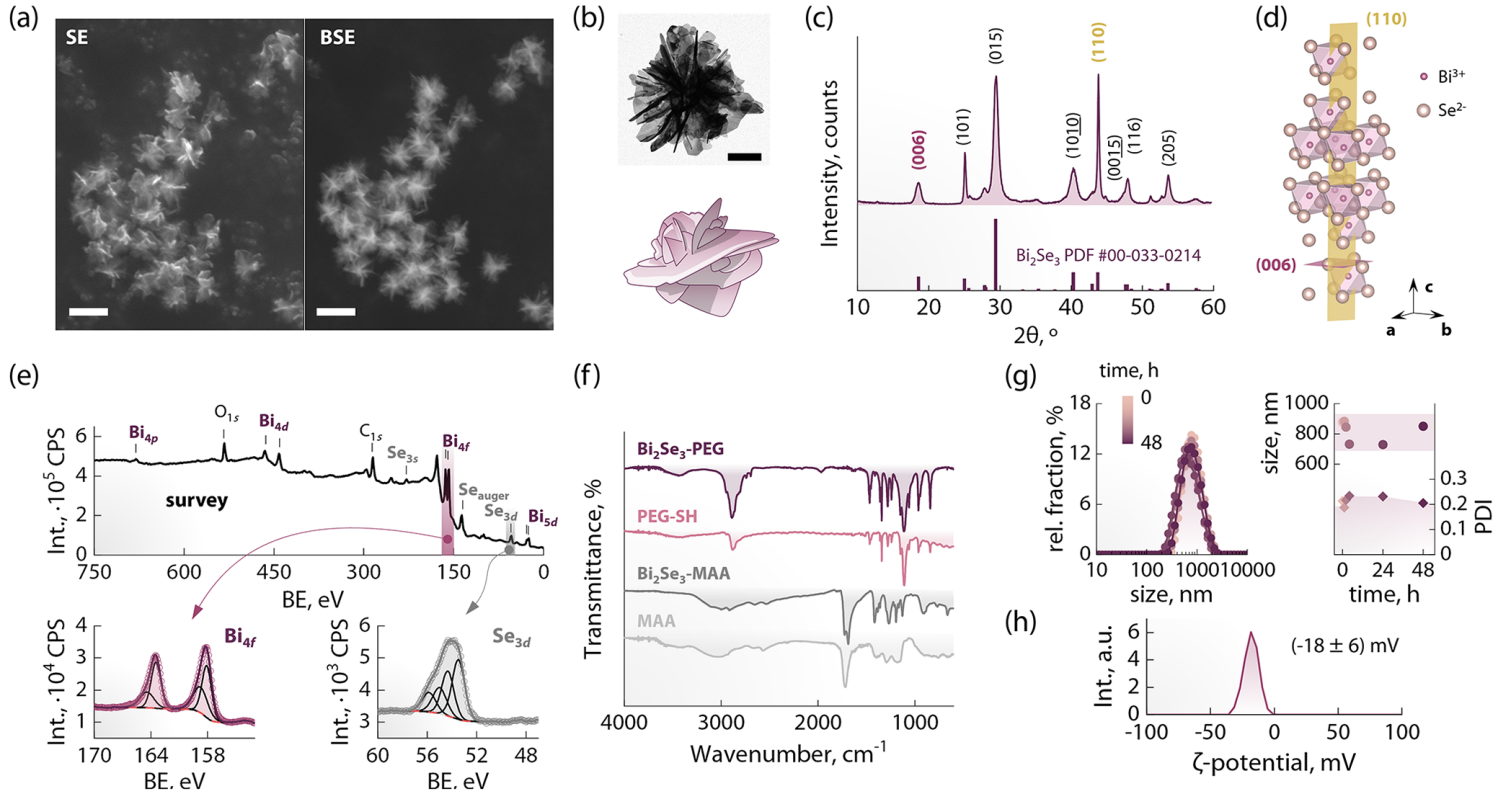
Physicochemical characterization of Bi_2_Se_3_ nanostructured clusters. (a) SEM image of the same area recorded in the SE (left) and BSE (right) mode. The reduced contrast in the BSE of the smaller structures visible in the SE (e.g., at the center right of the area) indicates that they are not made of Bi_2_Se_3_. The scale bars are 1 μm. (b) TEM image of an isolated Bi_2_Se_3_ cluster along with a sketch of the desert rose morphology. The scale bar is 200 nm. (c) PXRD pattern of the prepared sample (top) along with the reference pattern of Bi_2_Se_3_ (bottom, PDF #00-033-0214). (d) Unit cell of Bi_2_Se_3_ where two representative planes for the (110) and (006) families (in yellow and violet, respectively) are represented. (e) Survey (top) and high-resolution (bottom; Bi 4f left and Se 3d right) XPS spectra. BE stands for binding energy. (f) FTIR spectra of Bi_2_Se_3_ clusters before and after PEGylation, along with the spectra of pristine MAA and PEG-SH. (g) Hydrodynamic size distribution and PDI of PEGylated Bi_2_Se_3_ clusters measured in water over the course of 48 h. (h) ζ-Potential distribution of PEGylated Bi_2_Se_3_ clusters in water.

The herein developed microwave-assisted method allows producing Bi_2_Se_3_ nanostructured clusters that are already dispersed in water in minutes. This is in contrast with other methods for producing Bi_2_Se_3_, which entail the use of high-boiling solvents under an inert atmosphere^35^ (even in the case of microwave-assisted procedures^36^), or several hours of solvothermal reaction^37^ or ultrasonication^38^ (Table S1). The scanning electron microscopy (SEM) and transmission electron microscopy (TEM) observations of the synthesis product showed nanostructured clusters with a desert rose-like morphology, whose diameter was measured to be 810 ± 60 nm (Figures 1a,b and S1). Particularly insightful is the comparison of the SEM images obtained for the same area using secondary electron (SE mode) and back-scattered electron (BSE mode). In particular, the latter modality allows one to discern compositional differences and, in this case, makes it possible to clearly observe the different “petals” composing each structure—which are hardly observable in the SE image. The crystalline structure of these clusters was ascertained via PXRD, obtaining a diffraction pattern that matches the reference pattern of rhombohedral (*R*3̅*m*) Bi_2_Se_3_ (Figure 1c). As anticipated from the observed morphology of the clusters, composed of several two-dimensional “petals”, some reflections are much sharper than others. This is particularly true when comparing the family of planes characterized by low and high Miller indexes. It is common knowledge that the surface energy of low-index planes is lower, and thus preferential growth along those crystallographic directions is favored compared to high-index planes. In fact, applying the Scherrer equation to the (006) and (110) reflections (these planes are reported in Figure 1d), crystallite sizes of approximately 20 and 100 nm were found, respectively. These values agree well with the sizes observed in SEM and TEM images. To confirm the composition of the clusters, XPS measurements were also performed (Figure 1e).

From the survey spectrum, signals arising from bismuth and selenium were observed, along with oxygen and carbon. The other expected element is sulfur, which is present in the thiol group of MAA. However, its characteristic signals are found at 160–169 eV, hence overlapping with the signal from bismuth. Indeed, the high-resolution Bi 4f spectrum can be fitted with two doublets (Δ = 5.3 eV) whose more intense components are centered around 158.1 and 158.9 eV, respectively. The first contribution comes from bismuth bound to selenium (in the matrix)^39^ and to sulfur (in MAA), while the second likely stems from surface oxidation (BiO_*x*_). The Se 3d spectrum was also fitted with two doublets (Δ = 0.86 eV, the intensity ratio of 0.735). The most intense peaks fell at 53.5 and 55.0 eV, which could be assigned, respectively, to Se bound to Bi^39^ and, tentatively, to Se bound to S.

After the purification steps, the clusters feature on their surface MAA molecules, as confirmed using FTIR analysis (Figure 1f). However, their long-term colloidal stability in aqueous media is limited (see Figure S2). Therefore, MAA was exchanged for PEG-SH by simply adding the polymer to a water dispersion of the clusters. The dispersion immediately tuned more transparent and homogeneous. PEG-SH attachment was further promoted by ultrasonicating for few seconds. The success of this procedure was probed via FTIR, observing that after ligand exchange, the spectrum of the clusters showed the characteristic signals of PEG (Figure 1f). The mean hydrodynamic size of the Bi_2_Se_3_ clusters after PEGylation in water was monitored over the course of 48 h (Figure 1g). Its value fluctuated around 800 nm—compatibly with the size estimated from electron microscopy observations—and the polydispersity index (PDI) remained consistently below 0.25. These results indicate a lack of appreciable aggregation and/or a loss of integrity of the clusters. The negative ζ-potential of the clusters after PEG modification (−18 mV, Figure 1h) suggests that although PEG molecules are the preponderant species decorating the surface (as inferred from FTIR measurements), MAA molecules are still attached to the clusters. Indeed, this negative value likely stems from the deprotonation of exposed carboxylic groups found in MAA. The PEGylated Bi_2_Se_3_ clusters were also observed using scanning electron microscope, observing no noticeable changes in the structures (Figure S3). To confirm the reproducibility of the synthesis approach, three separate batches of Bi_2_Se_3_ clusters were prepared and PEGylated, followed by TEM observations (Figure S4). In all the three instances, the structures show the same desert rose morphology and are similar in size.

**Figure 2:**
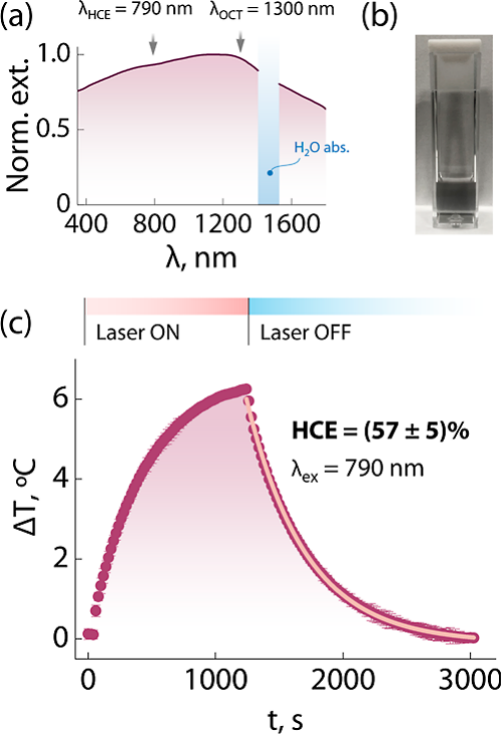
Optical properties of Bi_2_Se_3_ nanostructured clusters. (a) Extinction spectrum recorded on the Bi_2_Se_3_ cluster dispersion shown in (b). The wavelengths used for the determination of the HCE (790 nm) and the central wavelength of the OCT instrument (1300 nm) are indicated. (c) Heating–cooling cycle were recorded under the 790 nm excitation of a Bi_2_Se_3_ dispersion in water. The exponential fit of the cooling part is also plotted as a light pink solid line (*R*^2^ = 0.9994).

We then moved to investigate the optical properties of the Bi_2_Se_3_ clusters. This material is a topological insulator, meaning that its surface sustains electronic currents much like a metallic material, while its core behaves as an insulator. This configuration of the electronic states endows the Bi_2_Se_3_ clusters with strong light extinction capabilities throughout the explored wavelength range (350–1800 nm; Figure 2a), similarly to what was observed in previous work.^37^ The dark color of the dispersion is already a clear visual cue of the broadband photon extinction of the developed nanostructured clusters (Figure 2b), which covers the three biological windows (NIR-I: 750–950 nm; NIR-II: 1000–1350 nm; and NIR-III: 1500–1800 nm; see Figure 2a). This extended optical activity is pivotal in the biomedical context because it makes these materials applicable for imaging and therapeutic approaches that make use of very different NIR wavelengths. One of the possible scenarios is the use of Bi_2_Se_3_ clusters as photothermal agents in photothermal therapy. To that end, we estimated their photon-to-heat conversion efficiency (HCE) by adapting the approach initially introduced by Roper et al. (Figure 2c).^33,40^ The HCE retrieved from these measurements was 57 ± 5%, which was obtained considering the effective mass of the cuvette contributing to the heat dissipation^41^ (see Supporting Information, Figures S5 and S6 and Tables S2 and S3 for details about these calculations). This value is on par with the HCE reported for several other photothermal agents (Table S4). These results confirm the suitability of Bi_2_Se_3_ clusters as photothermal therapy agents, a concept already explored by other groups with nanoparticles having an analogous chemical composition.^42,43^ Furthermore, the obtained HCE value indicates that roughly 50% of the photons interacting with the Bi_2_Se_3_ clusters are absorbed (and later converted into heat), and thus 50% of the photons are instead scattered. This aspect is of fundamental importance for the performance of this nanomaterial as an OCT contrast agent (vide infra) because OCT contrast stems from the scattering of the probing photons.

**Figure 3:**
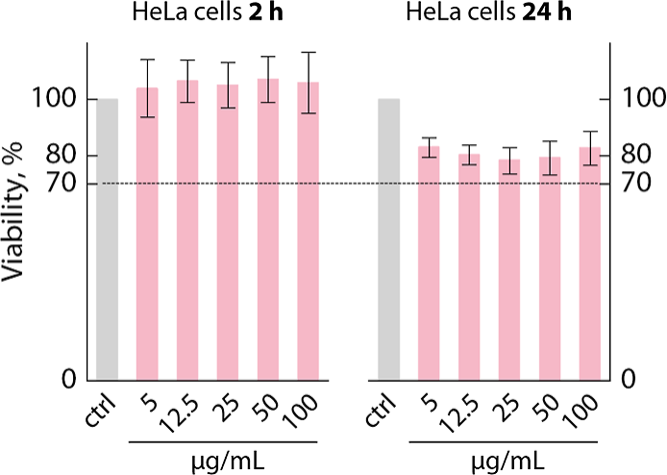
Results of the MTT test performed on HeLa cells to test the cytotoxicity of Bi_2_Se_3_ clusters. A dashed line is drawn at 70% of viability, the value indicated as the lower threshold in ISO 10993-5:2009 below which a compound is classified as having cytotoxic potential.

In order to assess the amenability of the developed Bi_2_Se_3_ clusters to be used in the biomedical context, in vitro cytotoxicity tests were performed, incubating HeLa cells with different concentrations of the particles (Figure 3). At the 2 h mark, no appreciable decrease in the viability was observed at any of the tested concentrations. After 24 h, a 20% decrease in viability occurred. Because according to ISO 10993-5:2009 a material is classified as having cytotoxic potential when the viability falls below 70% (dashed line in Figure 3), the Bi_2_Se_3_ clusters presented in this study could be considered biocompatible under tested conditions. These observations are in line with reports on the in vivo toxicity of Bi_2_Se_3_ plates of sizes up to 100 nm, which showed that their toxicity is low in mouse models.^30−32^ Given the large size of the reported Bi_2_Se_3_ clusters, one might have concerns about size-induced toxicity. To that end, it has been shown that the larger particles of different materials and shapes are mainly accumulated in the liver and spleen.^44−46^ Although generally no significant damage to the organs is reported after histological examination,^47,48^ size-related toxicity effects in vivo should be thoroughly examined before application of Bi_2_Se_3_ clusters at the clinical or preclinical stage.

**Figure 4:**
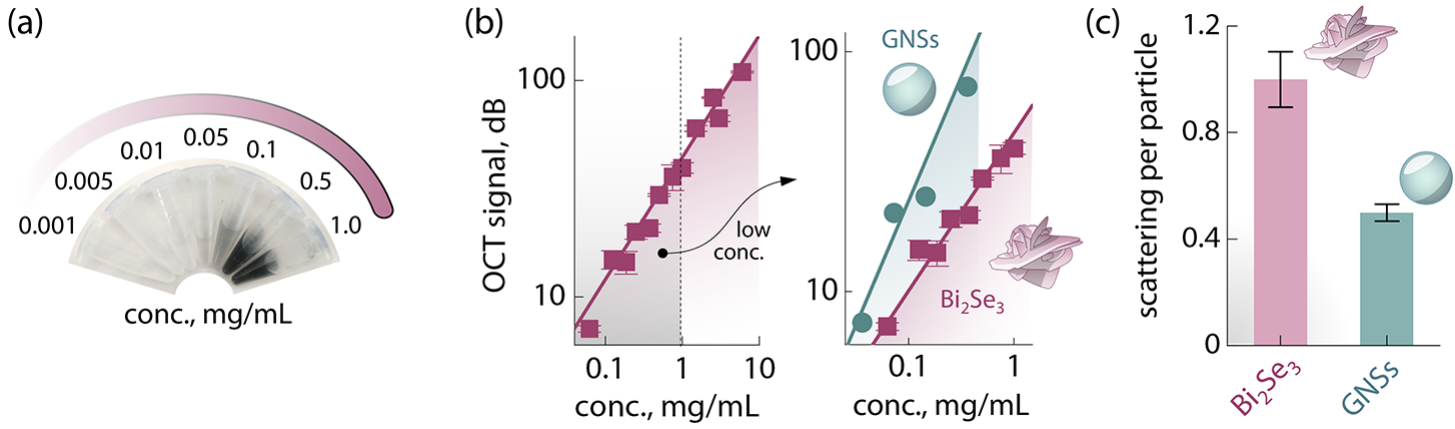
OCT performance of Bi_2_Se_3_ nanostructured clusters. (a) Photograph of representative Bi_2_Se_3_ cluster dispersions of different concentrations. (b) Log–log OCT calibration curve of the dispersions shown in (a) along with a zoom-in in the low-concentration range with a comparison of the performance of Bi_2_Se_3_ clusters with commercial GNSs. The lines are logarithmic fits to the experimental data. The error bars are obtained from two replicates of the measurements. (c) Normalized scattering per particle given by Bi_2_Se_3_ clusters and GNSs.

After testing the cytotoxicity of Bi_2_Se_3_ clusters, their prowess as OCT contrast agents was evaluated. For this purpose, several dilutions of the original Bi_2_Se_3_ cluster dispersion were prepared (Figure 4a). The calibration curve (i.e., the intensity vs concentration curve) of the Bi_2_Se_3_ clusters follows a logarithmic trend (Figure 4b) characteristic of the decibel (dB) scale used in the OCT systems, as previously reported for other systems.^18,27^ To benchmark the performance of Bi_2_Se_3_ clusters, their OCT signal in the low-concentration range was compared with the contrast given by GNSs—the commercially available particles with the best performance as OCT contrast agents (inset in Figure 4b). The developed Bi_2_Se_3_ clusters gave rise to an OCT signal that is of the same order of magnitude as the one generated by GNSs. Encouraged by this observation, we moved to compare the performance of the OCT contrast agents at the single-particle level, measuring the scattering per particle. This was accomplished by dividing the mean intensity measured over a region of interest in the OCT image by the total number of spots seen in that region. Intriguingly, Bi_2_Se_3_ clusters provided at least twice as much scattering per particle as GNSs (Figure 4c). This result can be explained in light of the overall larger size of the Bi_2_Se_3_ nanostructured clusters in hand with their photon extinction properties at the OCT working wavelength. Moreover, the desert rose morphology is expected to support a stronger interaction with the photons compared to simpler morphologies (e.g., plates and spheres), as observed in the similar flower-like “superstructures” of CuS used for photothermal therapy.^49^ The data provided in Figure 4b,c demonstrates that the performance of the Bi_2_Se_3_ clusters presented in this study as OCT contrast agents is similar to that of GNSs because both the total OCT signal and the scattering per particle of the two materials are in the same order of magnitude. These results were not unexpected, given the strong photon scattering capabilities of Bi_2_Se_3_ clusters, as deduced from the measured HCE value (vide supra).

**Figure 5:**
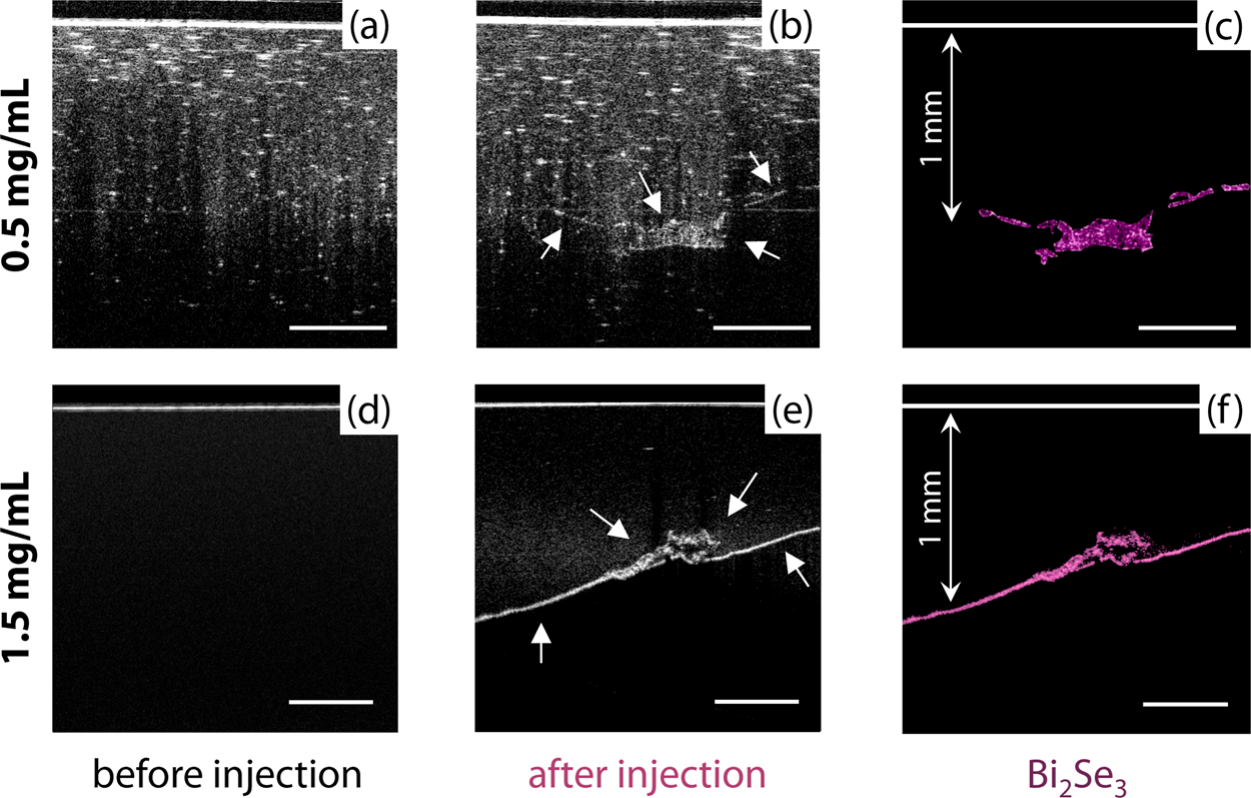
OCT images of an agar tissue phantom before (a,d) and after (b,e) an injection of a dispersion of Bi_2_Se_3_ clusters with a concentration of 0.5 and 1.5 mg/mL in PBS 1×. The arrows in b,e indicate the location of Bi_2_Se_3_ clusters. These zones were also highlighted in purple to better visualize them (c,f). The scale bars are 500 μm.

Lastly, the Bi_2_Se_3_ clusters were tested as contrast agents in Intralipid-based tissue phantoms, which mimic the optical behavior of biological tissues in the NIR both in terms of photon scattering and absorption. OCT images were taken before (Figure 5a,d) and within 5 min after (Figure 5b,c,e,f) injecting 50 μL of a 0.5 and 1.5 mg/mL dispersion of Bi_2_Se_3_ clusters in PBS 1×. A two and fourfold enhancement of the OCT intensity was observed in the regions where the clusters are located (highlighted in purple) compared to their surroundings. Note that Bi_2_Se_3_ clusters are readily observed at a tissue depth of 1 mm. Overall, these results indicate that the clusters presented in this study are good candidates as OCT contrast agents for deep-tissue imaging.

## Conclusions

We have herein presented, for the first time, Bi_2_Se_3_ nanostructured clusters as an inexpensive and easy-to-prepare alternative to GNSs as OCT contrast agents. These clusters were synthesized following a fast microwave-assisted procedure directly in water. The obtained clusters have a desert rose-like morphology, with several “petals” composing the overall structure. After modification of their surface with PEG, the clusters showed good size homogeneity, without the loss of integrity or aggregation when dispersed in water. The broad photon extinction of the Bi_2_Se_3_-based material extends into the NIR spectral range, covering the three biological windows. The measured HCE—close to 50%—indicates that roughly half of the impinging photons are scattered. This photon scattering capability is an important requisite for an effective OCT contrast agent. Moreover, cell viability tests confirmed that the Bi_2_Se_3_ clusters present low cytotoxicity, supporting their potential application for biomedical purposes. Lastly, in vitro studies revealed that the performance of these newly developed OCT contrast agents is comparable to that of GNSs (staple OCT contrast agents), both in terms of scattering intensity and the possibility to perform deep-tissue imaging.

It should be stressed that the prowess of Bi_2_Se_3_ clusters in the biomedical field is not restricted to bioimaging; indeed, their high HCE imbues them with good photothermal therapy capabilities, while the large X-ray absorption cross section of Bi^3+^ supports their use in X-ray-based imaging methodologies as well. Hence, the nanostructured materials herein presented have tangible potential as multimodal contrast agents and in theranostics. In addition, the high surface area featured by these clusters coupled with their strong interaction with photons over a broad wavelength range makes them of interest for applications in photocatalysis and hydrogen generation.

Future work will focus on the use of the developed contrast agents in photothermal OCT and their application in vivo upon modification of their surface to endow them with active targeting capabilities.

## Supporting Information Available

Overview of methods for the synthesis of Bi_2_Se_3_-based nanomaterials reported in the literature; additional SEM and TEM characterization of different Bi2Se3 clusters batches before and after PEGylation; details of the HCE determination; and comparison of the HCE values reported for other nanomaterials reported in the literature (PDF)
