# Supplementary material for: Bismuth Selenide Nanostructured Clusters as Optical Coherence Tomography Contrast Agents: Beyond Gold-Based Particles
Source: ACS Photonics. 2022 Feb 7;9(2):559–66. doi: 10.1021/acsphotonics.1c01504 (PMC8862561; doi:10.1021/acsphotonics.1c01504)
Supplement: Supplementary file 1 — ph1c01504_si_001.pdf [file ph1c01504_si_001.pdf]

Bismuth selenide nanostructured clusters as optical coherence tomography contrast agents: Beyond gold-based particles.

Jingke Yao,[a†] Tamara Muñoz Ortiz,[a†] Francisco Sanz-Rodríguez,[b,c] Emma Martín Rodríguez,[b,d] Dirk H. Ortgies,[a,b] José García Solé,[a,b] Daniel Jaque[a,b]* and Riccardo Marin[a]*

[a] Nanomaterials for Bioimaging Group (nanoBIG), Departamento de Física de Materiales, Facultad de Ciencias, Universidad Autónoma de Madrid, C/ Francisco Tomás y Valiente 7, Madrid 28049, Spain

[b] Nanomaterials for Bioimaging Group (nanoBIG), Instituto Ramón y Cajal de Investigación Sanitaria, Hospital Ramón y Cajal, Ctra. De Colmenar Viejo, Km. 9,100, 28034 Madrid, Spain

[c] Nanomaterials for Bioimaging Group (nanoBIG), Departamento de Biología, Facultad de Biología, Universidad Autónoma de Madrid, C/ Darwin 2, Madrid 28049, Spain

[d] Nanomaterials for Bioimaging Group (nanoBIG), Departamento de Física Aplicada, Facultad de Ciencias, Universidad Autónoma de Madrid, C/ Francisco Tomás y Valiente 7, Madrid 28049, Spain

TABLE OF CONTENT

1. Comparison of synthesis methods for Bi_2Se_3 nanomaterials S-2

2. Study of the morphology, size, reproducibility, and stability of Bi_2Se_3 clusters. S-3

3. Determination of the heat conversion efficiency of Bi_2Se_3 clusters. S-5

References S-8

1. Comparison of synthesis methods for Bi_2Se_3 nanomaterials

Table S1. Overview of the synthesis methods reported in the literature for the synthesis of Bi_2Se_3 nanomaterials. RT = room temperature.

Precursors	Reaction T	Reaction time	Solvent	Inert atm.	Morphology	Ref.
$Bi(NO_3)_3$, NaHSe	160 °C	10 min	ethylene glycol	N_2	nanosheet	1
$Bi(NO_3)_3$, Na_2SeO_3	140 °C	10 min	ethylene glycol	-	nanoplate	2
$Bi(NO_3)_3$, Na_2SeO_3	180 °C	10 min	ethylene glycol	N_2	nanodiscs	3
Na_2SeO_3, Bi_2O_3	150 °C	12 h	H_2O	-	hollow cavity structures	4
Bi_2O_3, Se	210 °C	24 h	ethylene glycol	-	nanoplates	5
$Bi(NO_3)_3$, Selenocysteine	80 °C	3 h	H_2O	-	flower-like and spherical shape	6
$Bi(NO_3)_3$, Na_2SeO_3	180 °C	1 min	ethylene glycol	-	nanosheets	7
$BiCl_3$, H_2SeO_3	RT	-	H_2O	-	nanobelt	8
$Bi(NO_3)_3$, Na_2SeO_3	RT	1 h	H_2O	-	rhombohedral	9
$Bi_2(Hsal)_3$, $SeCl_4$	RT	1h	H_2O	-	sphere-like shape	10
$Bi(NO_3)_3$, Se powder	RT	30 min	ethylene glycol	N_2	cucumber-like microparticles	11
$Bi(NO_3)_3$, Na_2SeO_3	180 °C	1 min	ethylene glycol	-	nanosheets	12
$Bi(NO_3)_3$, Na_2SeSO_3	RT	5 min	H_2O	-	irregular layered structures	13
**Bi_2O_3, SeO_2**	**180 °C**	**5 min**	**H_2O**	**-**	**nanostructured clusters**	**This study**

2. Study of the morphology, size, reproducibility, and stability of Bi_2Se_3 clusters.

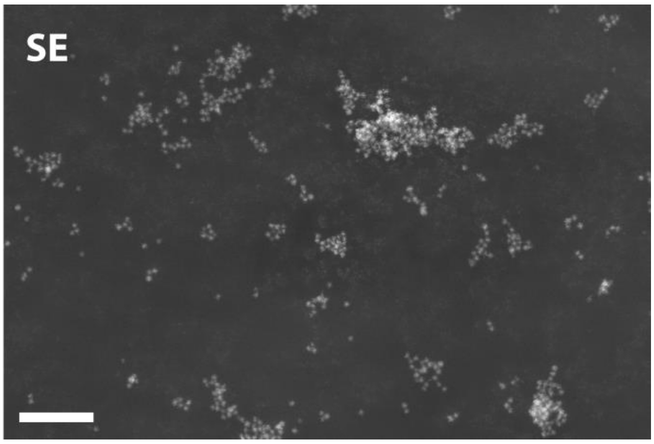

Figure S1. Secondary electron SEM image at low magnification (1.5 kx) of the MAA-coated Bi_2Se_3 clusters. Scale bar is 10 µm.

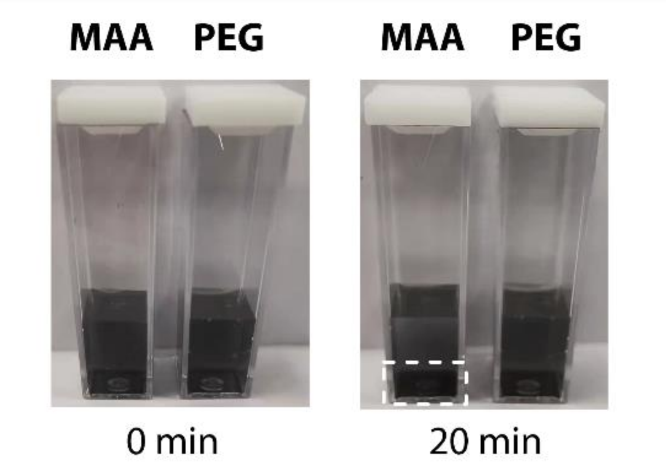

Figure S2. Comparison of the colloidal stability of Bi_2Se_3 clusters before and after PEGylation. It is evident MAA-coated clusters settle at a faster rate, as evidenced by the lighter color of the dispersion and the appearance of a dark sediment at the bottom of the cuvette (white, dashed rectangle in the rightmost figure).

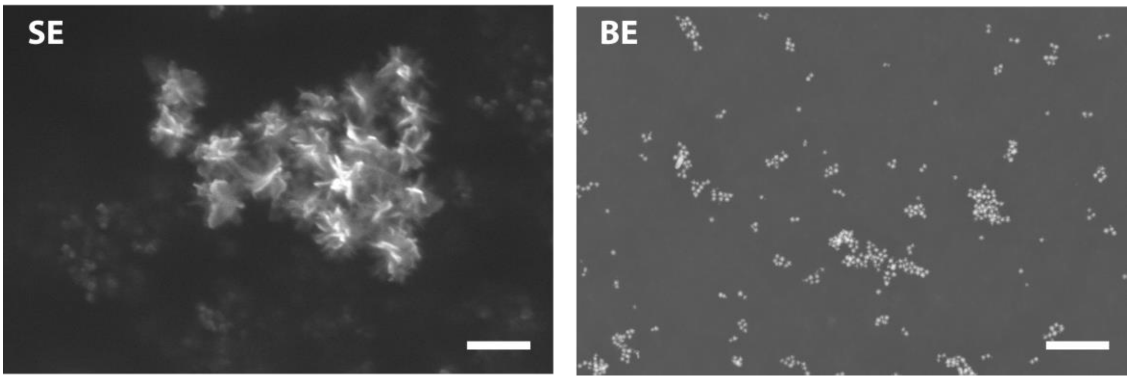

Figure S3. Secondary and back-scattered electron SEM images of pegylated Bi_2Se_3 clusters reported in Figure 1a and S1. Scale bars are 1 (left) and 10 (right) µm, respectively.

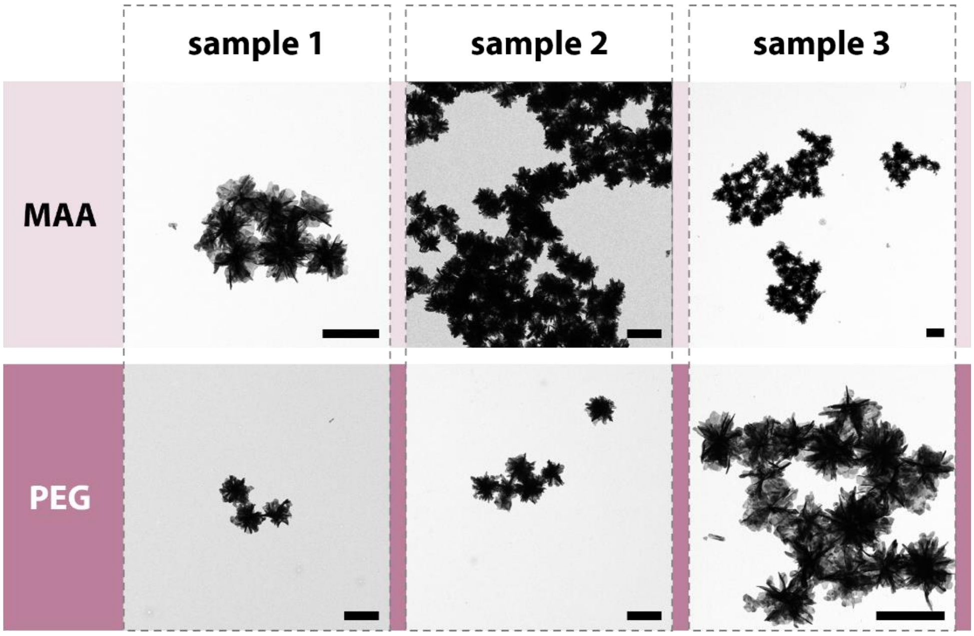

Figure S4. TEM images of three different batches of Bi_2Se_3 clusters before (MAA) and after (PEG) pegylation. Scale bars are 1 µm.

3. Determination of the heat conversion efficiency of Bi_2Se_3 clusters.

For the calculation of the heat conversion efficiency (HCE), we used the approach we employed for the determination of the HCE of CuS nanoparticles,[14] which is an adaptation of the original approach developed by Roper et al.[15]

Briefly, HCE is calculated as:

$$HCE = \frac{Q_{ext} - Q_0}{I(1 - 10^{-A_\lambda})}$$

Which contains the external heat injected into the dispersion of nanoparticles in the solvent (Q_{ext}), the heat dissipated by the solvent alone (Q_0), and an expression in the denominator which accounts for the radiated power extinguished by the nanoparticles alone (A_λ is the absorbance of the photon-to-heat converters at the excitation wavelength).

At the thermal equilibrium:

$$Q = hA(T - T_{\text{room}})$$

T_{room} is the room temperature and hA is the product of the heat transfer coefficient and the area of the sample in contact with the environment, calculated as:

$$hA = \frac{\sum_i m_i c_{p,i}}{\tau}$$

where m and c_p are the mass and heat capacity of the different materials that compose the sample, running through index i, and τ is the sample time constant, obtained by fitting the cooling part of the heating curves.

Because of the selected excitation wavelength (790 nm), which is poorly absorbed by water, aside from heating-cooling measurements (in triplicate) of a Bi_2Se_3 cluster dispersion in water and water under 790-nm excitation, a third measurement (also in triplicate) was performed with water under 980-nm excitation (**Figure S6**). The temperature increment for water was extracted from the measurement under 790-nm excitation, while the time constant (τ) was obtained from by fitting the cooling part of the heating-cooling curve under 980-nm excitation. Both temperature increment and time constant of Bi_2Se_3 clusters were obtained from the single set of measurements with 790-nm excitation.

Importantly, as we previously pointed out and recently Paściak et al.[16] confirmed, one should put care in considering the masses involved in the heat dissipation. Clearly, not all the cuvette is in contact with the liquid, and hence considering the whole mass of the cuvette would result in severe overestimation of the HCE. An "effective mass" could be considered instead. We here used a 1-cm optical glass cuvette with wall thickness of 0.125 cm, and a sample volume of 1 mL for all

experiments. We therefore considered that the mass of cuvette mainly involved in the heat exchange is the one in more direct contact with the fluid (**Figure S2**). This amounts to 1.665 g.

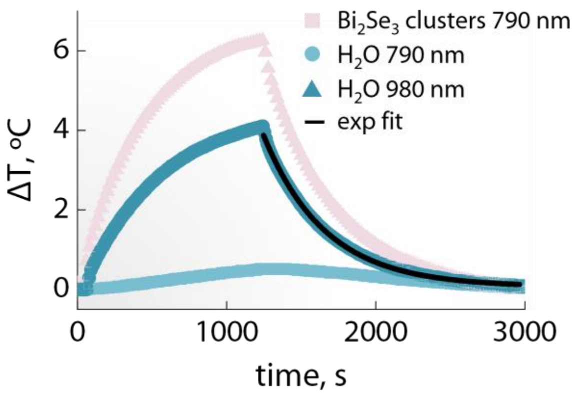

Figure S5. Heating-cooling curves obtained from the triplicated measurements of Bi_2Se_3 clusters dispersion in water (790 nm – light pink) and water (790 nm – light blue; 980 nm – dark blue). The exponential fit of the cooling part of the heating-cooling curve of water under 980-nm excitation is shown as a solid black line.

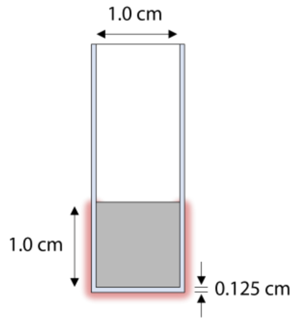

Figure S6. Sketch of the cuvette filled with Bi_2Se_3 cluster dispersion (in grey). The mass of the cuvette that was considered in the HCE calculations is highlighted with a red glow.

The values used for the HCE calculation are reported in **Table S2** and the parameters obtained from the fitting of the cooling part of the curves are reported in **Table S3**.

Table S2. Quantities used for calculating the HCE of Bi_2Se_3 nanostructured clusters.* Errors on the last digit are indicated within round parentheses.

	**ΔT (°C)**	**τ (s)**	**P_{790} (W)**	**A_{790}**	***Q***	**HCE**
H_2O	0.53(1)	413.9(7)	0.209(1)		0.007	
Bi_2Se_3	6.3(3)	465(1)	0.209(1)	0.348	0.072	0.57(5)

* $c_{p,\,water}$ = 4.186, $c_{p,\,glass}$ = 0.7

Table S3. Fitting parameters for the cooling part of H_2O (980 nm excitation) and Bi_2Se_3 (790 nm excitation) heating-cooling curves reported in **Figure S6** and **Figure 2c** of the main manuscript. The fitting function was of the form $y = y_0 + A_1 \cdot \exp(-x/\tau)$

	y_0	A_1	τ (s)	R^2
H_2O	0.066(2)	77.6(4)	413.9(7)	0.9994
Bi_2Se_3	-0.096(3)	88.1(5)	465(1)	0.9994

Table S4. Overview of some HCE values reported for nanomaterials proposed as photothermal agents.

Photothermal agent	λ_{ex} (nm)	Laser power (W)	HCE (%)	Ref.
Au nanorods/MoS_2	808	0.2	68.8	17
$Cu_{2-x}Se$	1064	1.56	13.2	18
gold nanorods/ graphene oxide	808	0.5	72.59	19
$TiSe_2$	808	1.0	65.58	20
$MnSe_2$	808	1.5	39.1	21
Gold nanorods/SiO_2	808	0.5	19.76	22
Gold−Platinum Nanodots	808	1	50.53	23
CuS	980	0.7	27.9	24
Gold nanorods	808	3.5	26	25
CuO/Cu_2O	1550	0.3	78	26
Au@Pt	808	1	55.8	27
MoO_2	808	0.8	62	28
hollow magnetite nanoclusters	1064	0.8	36.3	29
Pd	1064	0.69	33.98	30
gold nanorod@SiO_2@MnO_2	1064	0.4	27.47	31
**Bi_2Se_3 nanostructured clusters**	**790**	**0.209**	**57**	**This study**

References.

1 H. Xie, Z. Li, Z. Sun, J. Shao, X.-F. Yu, Z. Guo, J. Wang, Q. Xiao, H. Wang, Q.-Q. Wang, H. Zhang and P. K. Chu, Metabolizable Ultrathin Bi_2Se_3 Nanosheets in Imaging-Guided Photothermal Therapy *Small*, **2016**, *12,* 4136.

2 J. Li, F. Jiang, B. Yang, X.-R. Song, Y. Liu, H.-H. Yang, D.-R. Cao, W.-R. Shi and G.-N. Chen, Topological insulator bismuth selenide as a theranostic platform for simultaneous cancer imaging and therapy *Sci. Rep.*, **2013**, *3,* 1998.

3 Y. Min, G. D. Moon, B. S. Kim, B. Lim, J.-S. Kim, C. Y. Kang and U. Jeong, Quick, controlled synthesis of ultrathin Bi_2Se_3 nanodiscs and nanosheets *J. Am. Chem. Soc.*, **2012**, *134,* 2872.

4 C. Yang, M. Chang, M. Yuan, F. Jiang, B. Ding, Y. Zhao, P. Dang, Z. Cheng, A. A. A. Kheraif, P. Ma and J. Lin, NIR-Triggered Multi-Mode Antitumor Therapy Based on Bi_2Se_3/Au Heterostructure with Enhanced Efficacy *Small*, **2021**, *17,* 2100961.

5 Y. Liu, L. Cao, J. Zhong, J. Yu, J. He and Z. Liu, Synthesis of bismuth selenide nanoplates by solvothermal methods and its stacking optical properties *J. Appl. Phys.*, **2019**, *125,* 035302.

6 Y. You, J. Li, L. Chen, M. Wang, X. Dong, L. Yan, A. Zhang and F. Zhao, Photothermal Killing of A549 Cells and Autophagy Induction by Bismuth Selenide Particles *Materials*, **2021**, *14,* 3373.

7 G. Jia, Z. Wu, P. Wang, J. Yao and K. Chang, Morphological evolution of self-deposition Bi_2Se_3 nanosheets by oxygen plasma treatment *Sci. Rep.*, **2016**, *6,* 22191.

8 H. Liu, H. Cui, F. Han, X. Li, J. Wang and R. I. Boughton, Growth of Bi_2Se_3 Nanobelts Synthesized through a Co-Reduction Method under Ultrasonic Irradiation at Room Temperature *Cryst. Growth Design*, **2005**, *5,* 1711.

9 X. Qiu, J.-J. Zhu, L. Pu, Y. Shi, Y.-D. Zheng and H.-Y. Chen, Size-controllable sonochemical synthesis of thermoelectric material of Bi_2Se_3 nanocrystals *Inorg. Chem. Commun.*, **2004**, *7,* 319.

10 M. Ramezani, A. Sobhani-Nasab and A. Davoodi, Bismuth selenide nanoparticles: simple synthesis, characterization, and its light harvesting applications in the presence of novel precursor *J. Mater. Sci. Mater. Electron.*, **2015**, *26,* 5440.

11 R. Harpeness and A. Gedanken, Microwave-assisted synthesis of nanosized Bi_2Se_3 *New J. Chem.*, **2003**, *27,* 1191.

12 H. Xu, G. Chen, R. Jin, D. Chen, Y. Wang, J. Pei, Y. Zhang, C. Yan and Z. Qiu, Microwave-assisted synthesis of Bi_2Se_3 ultrathin nanosheets and its electrical conductivities *CrystEngComm*, **2014**, *16,* 3965.

13 A. D. Savariraj, V. Vinoth, R. V. Mangalaraja, T. Arun, D. Contreras, A. Akbari-Fakhrabadi, H. Valdés and F. Banat, Microwave-assisted synthesis of localized surface plasmon resonance enhanced bismuth selenide Bi_2Se_3 layers for non-enzymatic glucose sensing *J. Electroanal. Chem.*, **2020**, *856,* 113629.

14 R. Marin, A. Skripka, L. V. Besteiro, A. Benayas, Z. Wang, A. O. Govorov, P. Canton and F. Vetrone, Highly Efficient Copper Sulfide-Based Near-Infrared Photothermal Agents: Exploring the Limits of Macroscopic Heat Conversion *Small*, **2018**, *14,* 1803282.

15 D. K. Roper, W. Ahn and M. Hoepfner, Microscale Heat Transfer Transduced by Surface Plasmon Resonant Gold Nanoparticles *J. Phys. Chem. C.*, **2007**, *111,* 3636.

16 A. Paściak, A. Pilch-Wróbel, Ł. Marciniak, P. J. Schuck and A. Bednarkiewicz, Standardization of Methodology of Light-to-Heat Conversion Efficiency Determination for Colloidal Nanoheaters *ACS Appl. Mater. Interfaces*, **2021**,*13,* 44556.

17 M. R. Younis, C. Wang, R. An, S. Wang, M. A. Younis, Z.-Q. Li, Y. Wang, A. Ihsan, D. Ye and X.-H. Xia, Low Power Single Laser Activated Synergistic Cancer Phototherapy Using Photosensitizer Functionalized Dual Plasmonic Photothermal Nanoagents *ACS Nano,* **2019**, *13,* 2544.

18 X. Li, L. Yang, C. Men, Y. F. Xie, J. J. Liu, H. Y. Zou, Y. F. Li, L. Zhan and C. Z. Huang, Photothermal Soft Nanoballs Developed by Loading Plasmonic $Cu_{2-x}Se$ Nanocrystals into Liposomes for Photothermal Immunoassay of Aflatoxin B1 *Anal. Chem.*, **2019**, *91,* 4444.

19 M. R. Younis, R. B. An, Y.-C. Yin, S. Wang, D. Ye and X.-H. Xia, Plasmonic Nanohybrid with High Photothermal Conversion Efficiency for Simultaneously Effective Antibacterial/Anticancer Photothermal Therapy *ACS Appl. Bio Mater.*, **2019**, *2,* 3942.

20 Y. Duo, G. Luo, Z. Li, Z. Chen, X. Li, Z. Jiang, B. Yu, H. Huang, Z. Sun and X. Yu, Photothermal and Enhanced Photocatalytic Therapies Conduce to Synergistic Anticancer Phototherapy with Biodegradable Titanium Diselenide Nanosheets *Small*, **2021**, *17,* 2103239.

21 R. He, C. Ding, Y. Luo, G. Guo, J. Tang, H. Shen, Q. Wang and X. Zhang, Congener-Induced Sulfur-Related Metabolism Interference Therapy Promoted by Photothermal Sensitization for Combating Bacteria *Adv. Mater.*, **2021**, *33,* 2104410.

22 B. Zhang, H. Yan, Z. Meng, P. Li, X. Jiang, Z. Wu, J.-A. Xiao and W. Su, Photodynamic and Photothermal Ce6-Modified Gold Nanorod as a Potent Alternative Candidate for Improved Photoinactivation of Bacteria *ACS Appl. Bio Mater.*, **2021**, *4,* 6742.

23 S. Zhang, Q. Lu, F. Wang, Z. Xiao, L. He, D. He and L. Deng, Gold–Platinum Nanodots with High-Peroxidase-like Activity and Photothermal Conversion Efficiency for Antibacterial Therapy *ACS Appl. Mater. Interfaces*, **2021**, *13,* 37535.

24 R. Dai, X. Peng, B. Lin, D. Xu and R. Lv, NIR II Luminescence Imaging for Sentinel Lymph Node and Enhanced Chemo-/Photothermal Therapy for Breast Cancer *Bioconjugate Chem.*, **2021**, *32,* 2117.

25 Y. Sheng, M. Lin, X. Li, H. Hao, X. Lin, H. Sun and H. Zhang, Enhancement of the 808 nm Photothermal Effect of Gold Nanorods by Thiol-Induced Self-Assembly *Part. Part. Syst. Charact.*, **2014**, *31,* 788.

26 M. Shanmugam, N. Kuthala, R. Vankayala, C.-S. Chiang, X. Kong and K. C. Hwang, Multifunctional CuO/Cu_2O Truncated Nanocubes as Trimodal Image-Guided Near-Infrared-III Photothermal Agents to Combat Multi-Drug-Resistant Lung Carcinoma *ACS Nano*, **2021**, *15,* 14404.

27 Y. Bu, R. Huang, Z. Li, P. Zhang, L. Zhang, Y. Yang, Z. Liu, K. Guo and F. Gao, Anisotropic Truncated Octahedral Au with Pt Deposition on Arris for Localized Surface Plasmon Resonance-Enhanced Photothermal and Photodynamic Therapy of Osteosarcoma *ACS Appl. Mater. Interfaces,* **2021,** *13,* 35328.

28 Z. Sun, C. Wei, W. Liu, H. Liu, J. Liu, R. Hao, M. Huang and S. He, Two-Dimensional MoO_2 Nanosheet Composite Hydrogels with High Transmittance and Excellent Photothermal Property for Near-Infrared Responsive Actuators and Microvalves *ACS Appl. Mater. Interfaces*, **2021**, *13,* 33404.

29 X. Wang, C. Li, J. Qian, X. Lv, H. Li, J. Zou, J. Zhang, X. Meng, H. Liu, Y. Qian, W. Lin and H. Wang, Activatable Second Near-Infrared Fluorescent Probes: A New Accurate Diagnosis Strategy for Diseases *Small*, **2021**, *17,* 2100794.

30 M. Chang, Z. Hou, M. Wang, C. Yang, R. Wang, F. Li, D. Liu, T. Peng, C. Li and J. Lin, M. Chang, Z. Hou, M. Wang, C. Yang, R. Wang, F. Li, D. Liu, T. Peng, C. Li and J. Lin, Angew. Chem. Int. Ed., 2021, 60, 12971 *Angew. Chem. Int. Ed.*, **2021**, *60,* 12971.

31 T. He, C. Jiang, J. He, Y. Zhang, G. He, J. Wu, J. Lin, X. Zhou and P. Huang, Manganese-Dioxide-Coating-Instructed Plasmonic Modulation of Gold Nanorods for Activatable Duplex-Imaging-Guided NIR-II Photothermal-Chemodynamic Therapy *Adv. Mater.*, **2021**, *33,* 2008540.
